# Supplementary material for: Characteristics and Genomic Localization of Nurse Shark (Ginglymostoma cirratum) IgNAR
Source: Int J Mol Sci. 2024 Nov 29;25(23):12879. doi: 10.3390/ijms252312879 (PMC11641339; doi:10.3390/ijms252312879)
Supplement: Supplementary file 1 [file ijms-25-12879-s001.zip › ijms-3250951-supplementary.pdf]

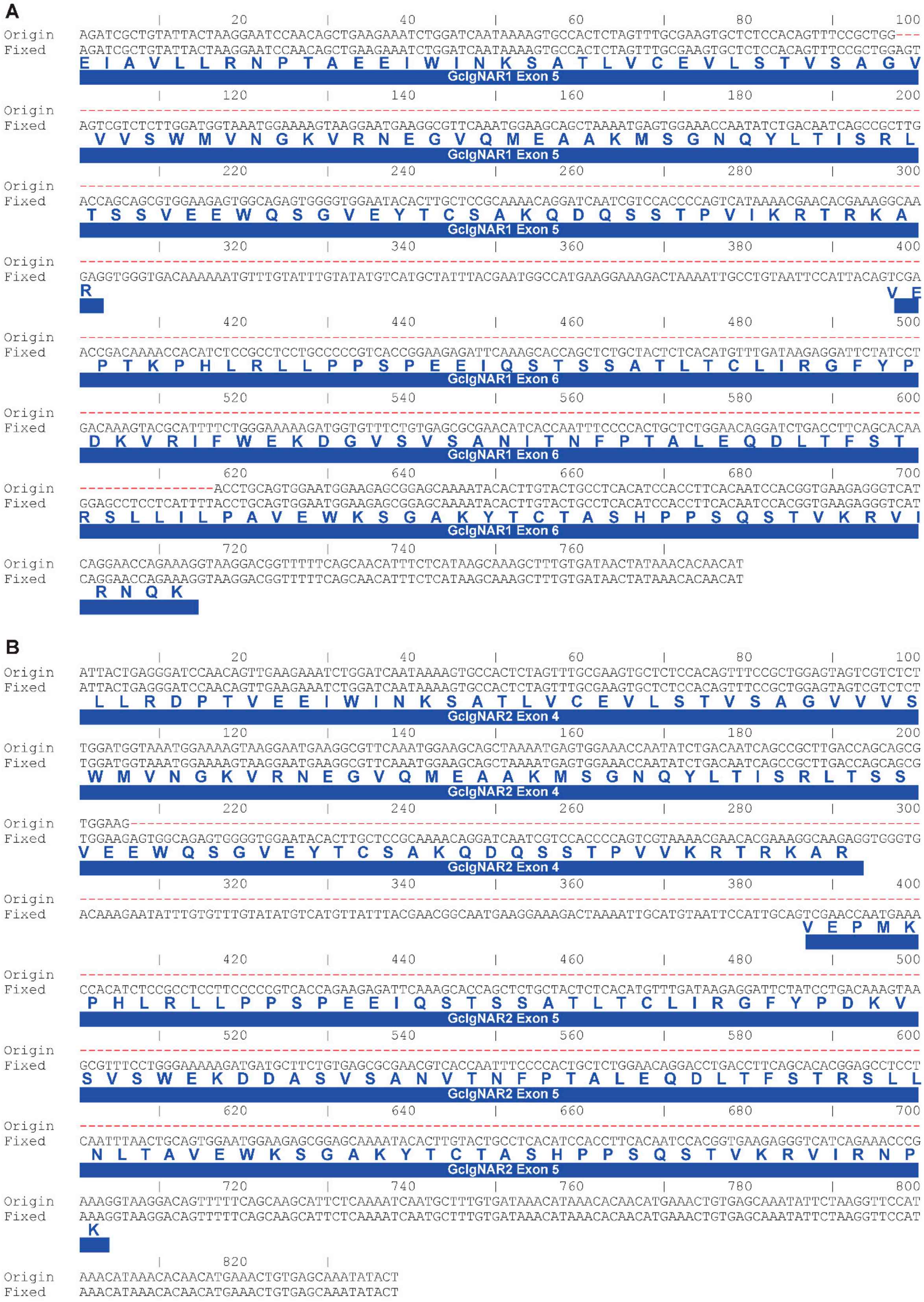
A
Origin
Fixed
GcIgNAR1 Exon 5
GcIgNAR1 Exon 6
B
GcIgNAR2 Exon 4
GcIgNAR2 Exon 5

Figure S1. Missing regions in the *GcIgNAR1* and *GcIgNAR2* loci. (**A-B**) The sequences of the missing regions in the *GcIgNAR1* (A) and *GcIgNAR2* (B) loci. The "Origin" label corresponds to the sequences from the released genome, while the "Fixed" label represents the sequences that have been corrected. The exons are labeled at the bottom of the amino acid sequences.

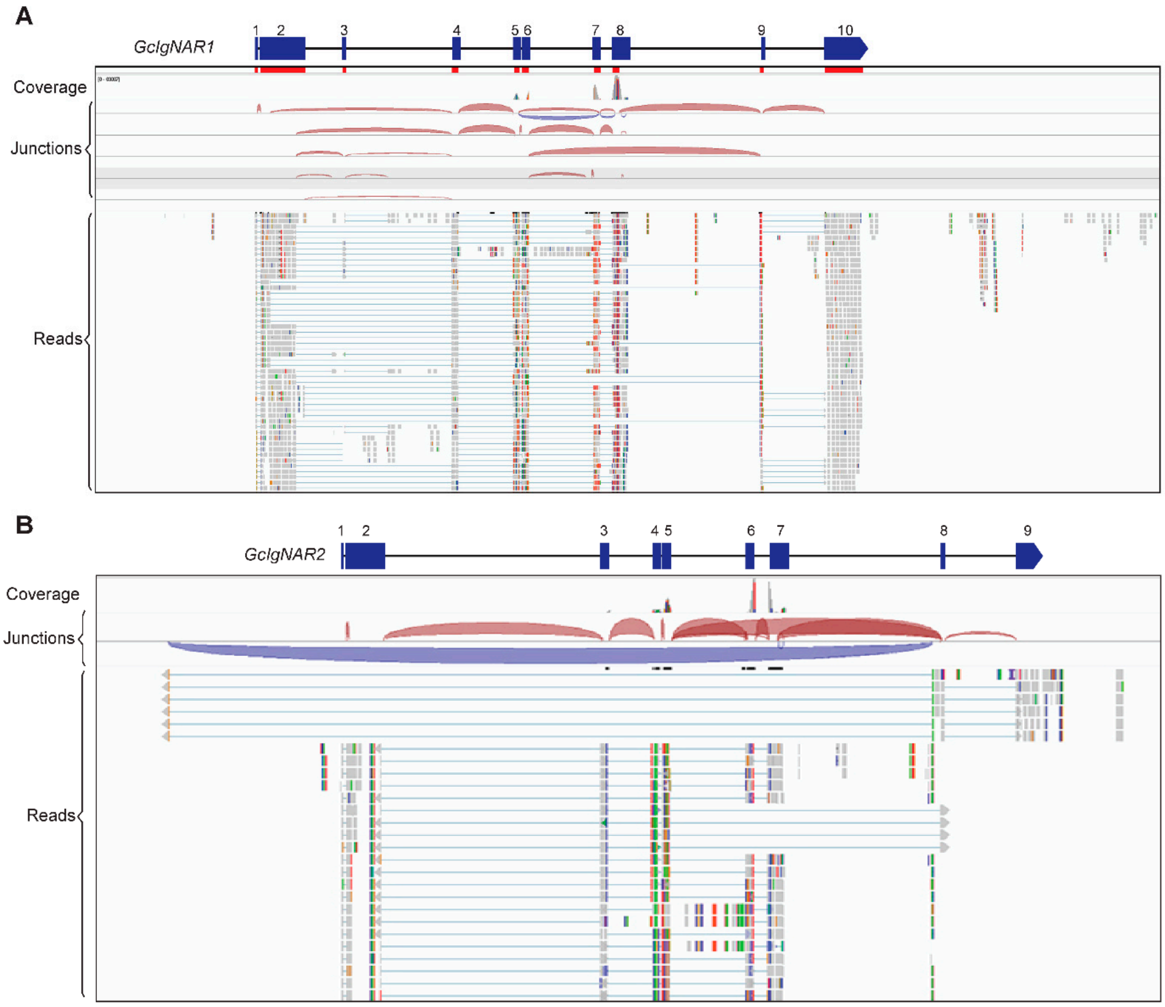

Figure S2. RNA splicing patterns of the *GcIgNAR1* and *GcIgNAR2* loci. (A-B) RNA splicing patterns of the *GcIgNAR1* (A) and *GcIgNAR2* (B) loci. The exons are shown at the top. The coverage, junctions, and read data are displayed below. The patterns of RNA splicing to generate the different isoforms are clearly observed through the junctions and read data in these loci.

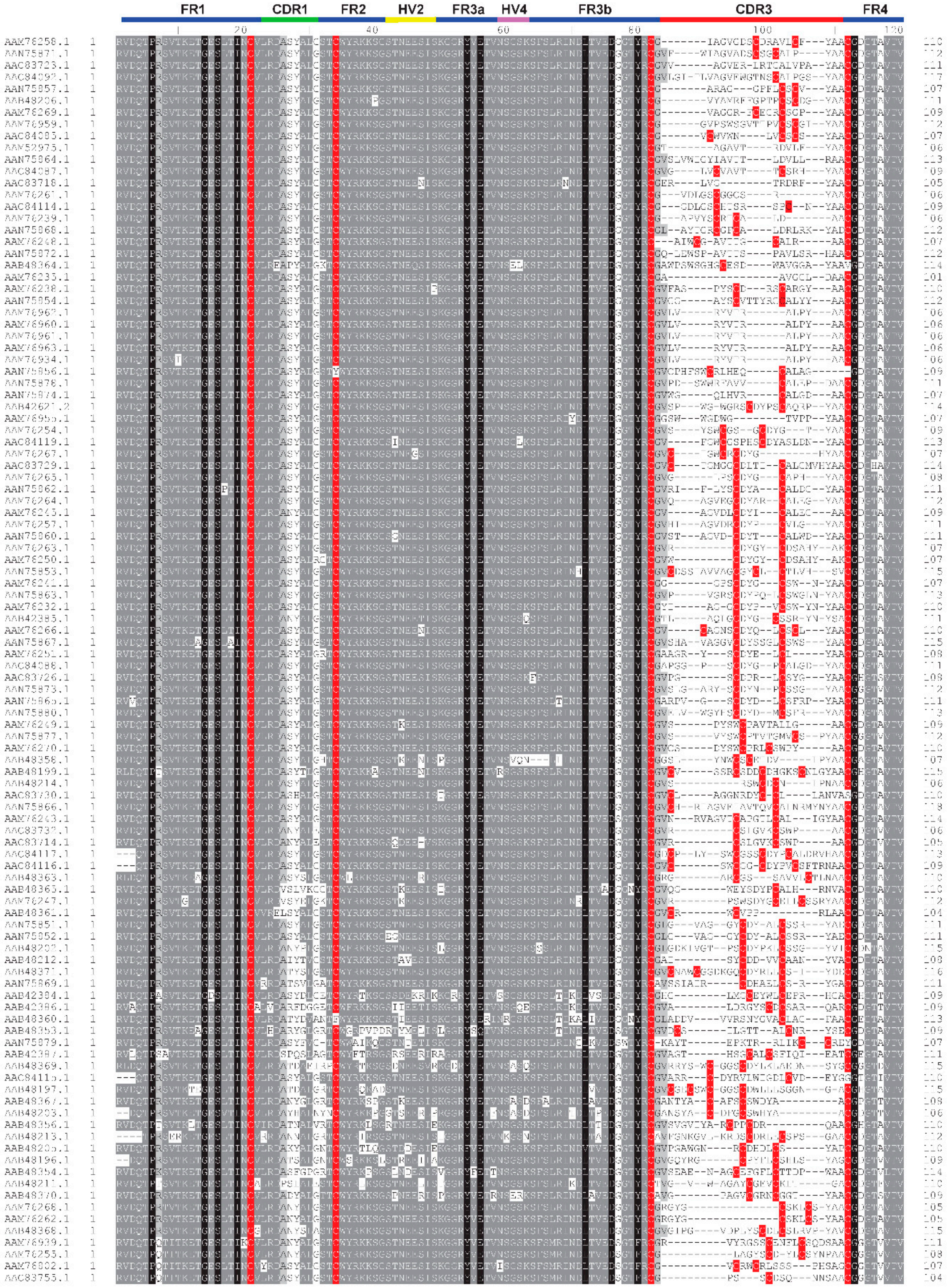

FR1
CDR1
FR2
HV2
FR3a
HV4
FR3b
CDR3
FR4
20
40
60
80
100
120

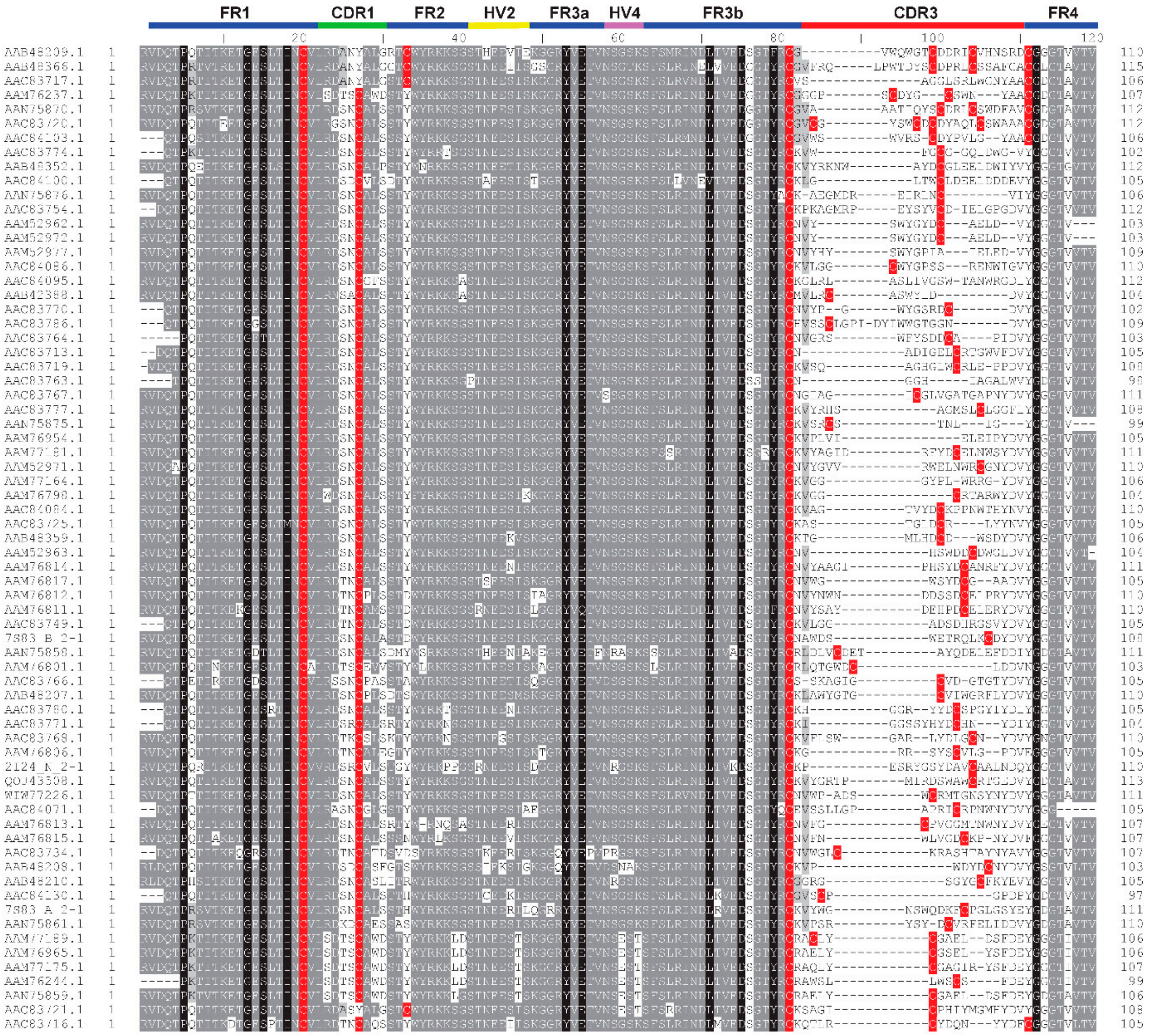

Figure S3. The alignment of the nurse shark VNAR sequences downloaded from NCBI database. Among these VNAR sequences, 109 sequences belong to GcIgNAR1 and 66 sequences belong to GcIgNAR2. The regions of VNAR are shown on the top of the sequences, and cysteines in FR1, CDR1, FR2 CDR3 and FR4 regions are highlighted with red.
